# Catatonia in a Patient With Bipolar Affective Disorder and Hypothyroidism: A Diagnostic and Therapeutic Challenge

**DOI:** 10.7759/cureus.46989

**Published:** 2023-10-13

**Authors:** Dillon M Woody, Charles Chen, Jonathan Parker

**Affiliations:** 1 Behavioral Health, Jackson Memorial Hospital, Miami, USA

**Keywords:** bush-francis catatonia rating scale, bipolar affective disorder, bipolar, hypothyroid, retarded catatonia

## Abstract

This case report presents the clinical course of a 33-year-old female with a history of bipolar affective disorder (BAD) who presented to the psychiatric emergency department with sudden-onset altered behavior, along with features indicative of catatonia. Before hospitalization, the patient had not been adherent to psychiatric medications for BAD for a period of several months, likely a contributing factor to the patient’s presenting symptoms. Over a two-week period before hospitalization, the patient exhibited progressive withdrawal, psychomotor retardation, disorganized behavior, and a lack of response to external stimuli. Initial labs upon admission had findings consistent with a diagnosis of hypothyroidism. The patient had no prior history of thyroid disease and further endocrinology workup was deferred by the hospitalist to outpatient care upon discharge.

While initially in the emergency department, the patient received intramuscular lorazepam for immediate symptom relief, the initial response to the Ativan challenge was not fully documented. Upon evaluation by the inpatient team the next morning, a Bush-Francis Catatonia Rating Scale score of 22 highlighted the severity of catatonia, which may have been further exacerbated by concurrent hypothyroidism. As such, thyroid hormone replacement therapy (levothyroxine) was indicated to normalize thyroid function. Combination treatment initially with lorazepam and levothyroxine was administered for the patient’s catatonia and olanzapine was chosen as the anti-psychotic.

Over the subsequent days, the patient’s catatonic symptoms demonstrated positive responses to treatment, prompting adjustments in pharmacotherapy. The patient eventually returned to baseline functioning, with substantial improvements in catatonia as well as mood symptoms.

This case underscores the complex interplay between catatonia, bipolar affective disorder, and thyroid dysfunction. The timely identification and management of hypothyroidism in the context of catatonia showcase the potential for favorable outcomes with targeted interventions.

## Introduction

Catatonia is a neuropsychiatric syndrome characterized by motor abnormalities, altered consciousness, and behavioral changes. It can be further defined as a behavioral syndrome characterized by an inability to move normally despite the capacity to do so [1]. It can occur in various psychiatric disorders, including bipolar affective disorder (BAD). The association between catatonia and BAD is well-established, but the role of underlying medical conditions, such as hypothyroidism, in precipitating catatonia remains an area of interest.

Hypothyroidism is known to have neuropsychiatric manifestations and has been associated with the development of catatonic symptoms [2]. However, the link between hypothyroidism and catatonia in patients with BAD is not well understood. This case report aims to shed light on this complex relationship and highlight the importance of considering medical factors in the etiology and management of catatonia in patients with underlying psychiatric conditions.

The patient’s presentation of psychosis, depressive features, catatonia, and hypothyroidism prompted a treatment course including lorazepam, clonazepam, olanzapine, and levothyroxine that showed beneficial treatment response.

## Case presentation

A 33-year-old female with a history of BAD was brought by her family to the psychiatric emergency department (ED) with the onset of altered behavior. Over a two-week period before hospitalization, the patient’s family reported that the patient had become increasingly withdrawn, demonstrating diminished speech, psychomotor retardation, disorganized behavior, and a lack of response to external stimuli. Our patient had a prior hospitalization several months before at a different hospital, then diagnosed with bipolar disorder and was prescribed risperidone. The family indicated that the patient had not wanted to follow up with a psychiatrist and had not taken medications for the last several months before her current hospitalization, likely a precipitating factor for our patient’s presentation. Labs collected upon admission were concerning for hypothyroidism, and after consultation with the inpatient hospitalist team, a new diagnosis of hypothyroidism was made, and treatment began.

On arrival at the crisis ED, the patient was selectively mute, staring into space, and immobile, features suggestive of catatonia. Vitals obtained in the ED displayed a blood pressure of 131/90 mmHg, a respiratory rate of 18 breaths/minute, a pulse rate of 79 beats/minute, and an oral temp of 36.7°C (low). Laboratory studies of a urinalysis obtained from the patient had returned negative for illicit substances or alcohol. Laboratory investigations confirmed the presence of hypothyroidism, showing elevated thyroid-stimulating hormone (TSH) levels at 35.530 µIU/mL and reduced free thyroxine (T4) levels at 0.76 ng/dL (Table 1).

**Table 1:** Thyroid panel. TSH = thyroid-stimulating hormone; T4 = thyroxine

Thyroid hormone	Patient’s level	Normal range
TSH (µIU/mL)	35.530	0.4–4.0
T4 (ng/dL)	0.76	4.5–11.2

Upon admission and while still in the ED behavioral health crisis unit, the patient was administered an intramuscular lorazepam (2 mg) challenge for immediate symptom relief, which, although minimal, resulted in improvement in her catatonic features. Intramuscular lorazepam was provided as no intravenous (IV) access was possible while the patient was in the behavioral health unit. However, the response was not sustained, warranting further interventions. During her first morning rounds with the inpatient psychiatric team on 7/26/2023, the patient presented with staring, mutism, and immobility; she was able to nod her head yes or no for the symptoms she was experiencing. The patient was able to follow some motor commands; however, they were unable to respond with verbal answers to questions. The patient scored a 22 on the Bush-Francis Catatonia Rating Scale (BFCRS). Given the severity of catatonia and its potential association with hypothyroidism, the patient was initiated on thyroid hormone replacement therapy (levothyroxine 50 µg every morning) to normalize her thyroid function in conjunction with a lorazepam challenge 0.5 mg oral TID with an initial plan to titrate over the next day depending upon observed response.

On day three of admission (07/27/2023), the patient showed minimal improvement in catatonia symptoms, scoring a 15 on the BFCRS. The patient continued to show symptoms of catatonia including mutism, complete withdrawal, fixed gaze or closed eyes, and psychomotor retardation with minimal posturing. The patient maintained the same position in bed, with a notable absence of rigidity and waxy flexibility. The patient provided no resistance to movement with commands. The decision was made to increase the dose of lorazepam to 1 mg oral TID. On day three, the patient was also started on olanzapine 5 mg PO daily for the treatment of psychosis (Table 2). On day four of admission (07/28/23), the patient showed marked improvement with a score of 7 on the BFCRS. The patient was withdrawn, but able to stand and engage in slow speech. The patient was still psychotic with poor organization and insight. The patient was able to participate in group activities with staff and other patients. A treatment decision was made to discontinue the lorazepam challenge and switch to a longer-acting benzodiazepine, i.e., clonazepam 0.5 mg oral TID. Over the next few days, the patient returned to her baseline level of functioning and endorsed improvements in her mood, psychosis, and catatonia symptoms. The final score on the BFCRS was 2/69 on the day of inpatient hospital discharge. See Table 2 for a chronologic comparison of the treatment regimen compared to BFCRS.

**Table 2:** Chronologic administration of medications and Bush-Francis Catatonia Rating Scale. PO: per os (by mouth). This abbreviation indicates that the medication is taken orally. IM: intramuscular. This abbreviation indicates that the medication is administered via injection into a muscle. TID: three times a day. This abbreviation indicates that the medication is taken three times throughout the day. mg: milligrams. This unit of measurement is used to denote the dosage of medications. µg: micrograms. This unit of measurement is used to denote smaller quantities of medications. am: ante meridiem (morning). This abbreviation indicates that the medication is administered in the morning. pm: post meridiem (afternoon/evening). This abbreviation indicates that the medication is administered in the afternoon or evening. Bedtime: refers to the time at which the medication is taken before going to sleep.

Treatment day	Day 1 07/25/23	Day 2 07/26/23	Day 3 07/27/23	Day 4 07/28/23	Day 07/29/23	Day 6 07/30/23	Day 7 07/31/23	Day 8 08/01/23	Day 9 08/02/23
Levothyroxine	Negative	Negative	50 µg PO at breakfast	50 µg PO at breakfast	50 µg PO at breakfast	50 µg PO at breakfast	50 µg PO at breakfast	50 µg PO at breakfast	50 µg PO at breakfast
Lorazepam	2 mg IM 23:33 pm	0.5 mg PO at 13:39 pm	0.5 mg PO at 08:47 am	1 mg PO at 09:38 am	Negative	Negative	Negative	Negative	Negative
		0.5 mg PO at 21:22 pm	1 mg PO at 16:32 pm						
			1 mg PO at 21:11 pm						
Olanzapine	Negative	Negative	5 mg PO 16:32 pm	5 mg PO at 09:38 am	5 mg PO at breakfast	5 mg PO at breakfast	5 mg PO at bedtime	5 mg PO at bedtime	Negative
Clonazepam	Negative	Negative	Negative	0.5 mg PO 14:30 pm	0.5 mg PO TID	0.5 mg PO TID	0.5 mg PO TID	0.5 mg PO TID	0.5 mg PO TID
				0.5 mg PO 21:15 pm					
Bush-Francis Catatonia Rating Scale	Not performed	22/69	15/69	7/69	6/69	4/69	4/69	4/69	2/69

## Discussion

This case report describes a 33-year-old female with a history of BAD who presented with sudden-onset altered behavior, culminating in catatonic features, and subsequent diagnosis of hypothyroidism. The patient’s unique clinical presentation raises important considerations regarding the complex relationship between catatonia, BAD, and thyroid dysfunction. Over the course of the patient’s nine-day hospitalization, the patient demonstrated significant improvement, with a resolution of catatonic features, improved thought process and verbal communication, and increased responsiveness to external stimuli. Her mood stabilized, and she displayed greater interest in her surroundings.

A noteworthy aspect of this case is the possibility of nonadherence with medication for BAD, which may have been a possible cause for the development of psychosis and catatonia. Medication nonadherence is a common challenge in the management of chronic psychiatric illnesses and can lead to exacerbation of symptoms, including catatonia [3]. This highlights the importance of patient education, regular follow-ups, and strategies to improve adherence to treatment regimens in the management of BAD.

The identification of elevated TSH levels and reduced T4 levels in this patient suggests a new onset of hypothyroidism, which may have contributed to the development of catatonic symptoms. Hypothyroidism is known to have neuropsychiatric manifestations, and its association with catatonia has been previously reported [2]. Thyroid hormone plays a crucial role in maintaining neurological and psychological functioning, and disturbances in thyroid function can lead to psychiatric symptoms, including catatonia [4,5].

The initial management of catatonic symptoms with intramuscular low-dose lorazepam resulted in minimal improvement, prompting the need for increased dosing. The patient’s score on the BFCRS demonstrated the severity of catatonia, guiding treatment decisions. Initiation of thyroid hormone replacement therapy (levothyroxine) aimed to normalize thyroid function and address the potential association between hypothyroidism and catatonia. Concomitant administration of lorazepam and levothyroxine allowed for a multi-pronged approach to symptom relief.

Treatment response in catatonia is usually one of complete resolution. Benzodiazepines and electroconvulsive therapy are the current recommended treatment options available [6]. In a study conducted by Bush and colleagues, it was reported that 2 mg IV lorazepam reduced catatonia scores on the BFCRS by 60% within 10 minutes [7,8]. However, there is still very limited statistical data in regard to patients with hypothyroidism and catatonia.

The greatest improvement in the patient’s catatonia symptoms occurred between day three and day four. On day three of admission, levothyroxine was added to the treatment plan, along with a concurrent increase in the dose of lorazepam; increased from 0.5 mg oral TID to 1 mg oral TID. The marked improvement in patient status may likely be attributed to the lorazepam administration due to a more rapid onset of action compared to levothyroxine. When taken orally, lorazepam typically starts to take effect within 30 to 60 minutes after ingestion [9]. On average, the half-life of lorazepam is about 10 hours in healthy adults [9].

Levothyroxine, also known as L-thyroxine or T4, is a synthetic form of the thyroid hormone thyroxine, which is used to treat hypothyroidism. Regarding the time it takes for an oral dose of levothyroxine to have an effect, it is important to understand that levothyroxine is a long-acting medication with a gradual onset of action. After taking an oral dose of levothyroxine, it needs to be absorbed in the gastrointestinal tract and then converted to its active form (triiodothyronine or T3) in the body’s tissues. It may take weeks for individuals to experience the full therapeutic effects of levothyroxine. The half-life of levothyroxine can vary depending on individual factors such as age, health status, and metabolism. On average, the half-life of levothyroxine is approximately seven days in healthy individuals [10].

Transition to clonazepam and olanzapine

Our patient was transitioned to clonazepam, a longer-acting benzodiazepine, in an effort to minimize potential discontinuation symptoms in the future when tapering the benzodiazepine dose. Olanzapine likely contributed to the improvement of our patient’s thought process, delusions, and cognitive deficits resulting from psychosis. The combination of psychopharmacological agents tailored to the patient’s presentation emphasizes the importance of individualized treatment strategies in managing complex cases of catatonia.

## Conclusions

This case highlights the significance of considering multiple contributing factors when encountering patients with catatonia and a history of BAD. It emphasizes the importance of conducting a thorough medical evaluation, including investigations for thyroid dysfunction, to ascertain any underlying medical conditions that may influence the course and treatment response. The timely identification and management of new-onset hypothyroidism in the context of catatonia demonstrate the potential for favorable outcomes with appropriate interventions. Collaboration between psychiatric and medicine teams is paramount in delivering comprehensive care to patients with overlapping clinical features.

In conclusion, this case report highlights the need for heightened awareness of catatonia in patients with BAD and highlights the potential association with new-onset hypothyroidism. It adds to the growing body of literature on the complexities of catatonia and its relationship with underlying medical conditions, offering insights into diagnostic and therapeutic considerations. Further research is warranted to elucidate the mechanistic link between catatonia, BAD, and thyroid dysfunction, as well as to optimize treatment strategies for patients with similar presentations.

## References

[1] Fink M, Taylor MA (2009). The catatonia syndrome: forgotten but not gone. Arch Gen Psychiatry.

[2] Asnis GM (2020). Catatonia secondary to hypothyroidism may be very responsive to electroconvulsive therapy: a case study. J ECT.

[3] Sajatovic M, Bauer MS, Kilbourne AM, Vertrees JE, Williford W (2006). Self-reported medication treatment adherence among veterans with bipolar disorder. Psychiatr Serv.

[4] Leigh H, Kramer SI (1984). The psychiatric manifestations of endocrine disease. Adv Intern Med.

[5] Carroll BT, Kennedy JC, Goforth HW (2000). Catatonic signs in medical and psychiatric catatonias. CNS Spectr.

[6] Francis A (2010). Catatonia: diagnosis, classification, and treatment. Curr Psychiatry Rep.

[7] Bush G, Fink M, Petrides G, Dowling F, Francis A (1996). Catatonia. I. Rating scale and standardized examination. Acta Psychiatr Scand.

[8] Bush G, Fink M, Petrides G, Dowling F, Francis A (1996). Catatonia. II. Treatment with lorazepam and electroconvulsive therapy. Acta Psychiatr Scand.

[9] Hui D (2018). Benzodiazepines for agitation in patients with delirium: selecting the right patient, right time, and right indication. Curr Opin Support Palliat Care.

[10] Colucci P, Yue CS, Ducharme M, Benvenga S (2013). A review of the pharmacokinetics of levothyroxine for the treatment of hypothyroidism. Eur Endocrinol.

